# *In vitro* and *in silico* evaluation of the design of nano-phyto-drug candidate for oral use against *Staphylococcus aureus*

**DOI:** 10.7717/peerj.15523

**Published:** 2023-06-08

**Authors:** Yasemin Budama-Kilinc, Bahar Gok, Cigdem Cetin Aluc, Serda Kecel-Gunduz

**Affiliations:** 1 Bioengineering Department, Yildiz Technical University, Istanbul, Turkey; 2 Health Biotechnology Joint Research and Application Center of Excellence, Istanbul, Turkey; 3 Graduate School of Natural and Applied Science, Yildiz Technical University, Istanbul, Turkey; 4 Abdi Ibrahim Production Facilities, Abdi Ibrahim Pharmaceuticals, Istanbul, Turkey; 5 Physics Department, Istanbul University, Istanbul, Turkey

**Keywords:** *O. acanthium* extract, PLGA nanoparticle, Antibacterial activity, Mutagenicity, Cytotoxicity, Molecular docking, MD

## Abstract

*Onopordum acanthium* is a medicinal plant with many important properties, such as antibacterial, anticancer, and anti-hypotensive properties. Although various studies reported the biological activities of *O. acanthium*, there is no study on its nano-phyto-drug formulation. The aim of this study is to develop a candidate nano-drug based on phytotherapeutic constituents and evaluate its efficiency *in vitro* and *in silico*. In this context, poly (lactic-co-glycolic acid) (PLGA) nanoparticles (NPs) of *O. acanthium* extract (OAE) were synthesized and characterized. It was determined that the average particle size of OAE-PLGA-NPs was 214.9 ± 6.77 nm, and the zeta potential was −8.03 ± 0.85 mV, and PdI value was 0.064 ± 0.013. The encapsulation efficiency of OAE-PLGA-NPs was calculated as 91%, and the loading capacity as 75.83%. The *in vitro* drug release study showed that OAE was released from the PLGA NPs with 99.39% over the 6 days. Furthermore, the mutagenic and cytotoxic activity of free OAE and OAE-PLGA-NPs were evaluated by the Ames test and MTT test, respectively. Although 0.75 and 0.37 mg/mL free OAE concentrations caused both frameshift mutation and base pair substitution (*p* < 0.05), the administered OAE–PLGA NP concentrations were not mutagenic. It was determined with the MTT analysis that the doses of 0.75 and 1.5 mg/mL of free OAE had a cytotoxic effect on the L929 fibroblast cell line (*p* < 0.05), and OAE-PLGA-NPs had no cytotoxic effect. Moreover, the interaction between the OAE and *S. aureus* was also investigated using the molecular docking analysis method. The molecular docking and molecular dynamics (MD) results were implemented to elucidate the *S. aureus* MurE inhibition potential of OAE. It was shown that quercetin in the OAE content interacted significantly with the substantial residues in the catalytic pocket of the *S. aureus* MurE enzyme, and quercetin performed four hydrogen bond interactions corresponding to a low binding energy of −6.77 kcal/mol with catalytic pocket binding residues, which are crucial for the inhibition mechanism of *S. aureus* MurE. Finally, the bacterial inhibition values of free OAE and OAE–PLGA NPs were determined against *S. aureus* using a microdilution method. The antibacterial results showed that the inhibition value of the OAE–PLGA NPs was 69%. In conclusion, from the *in vitro* and *in silico* results of the nano-sized OAE-PLGA NP formulation produced in this study, it was evaluated that the formulation may be recommended as a safe and effective nano-phyto-drug candidate against *S. aureus*.

## Introduction

*Staphylococcus aureus* is a dangerous organism that is a major cause of bacterial infections in community settings and hospitals (Carter et al., 2020). This pathogen is known to be more strongly associated with mortality than other bacterial pathogens. *S. aureus* can enter the bloodstream through cuts or open wounds in the skin, epithelium, or mucosal surface (McCaig et al., 2006). This leads to dangerous diseases, such as skin tissue infections, soft tissue infections, and debilitating and often fatal infections of the blood, bones, brain, and vital internal organs (Crossley et al., 2009; Labreure, Sona & Turos, 2019). For example, *S. aureus*, a deadly bacterium, causes bacterial abscesses in the body, such as endocarditis and lung infections, which can lead to a patient’s death from heart failure (Sibbald et al., 2006).

Conventional antibiotics are available for treating *S. aureus* infections. However, multidrug-resistant strains of *S. aureus* are a major health hazard for humans and economic burden for governments because they are lethal (Barman et al., 2016; Wang et al., 2017; Yang et al., 2017). Additionally, there are many problems with the use of conventional antibacterial drugs, such as low water solubility and stability, low oral bioavailability, frequent drug administration, and toxicity (Barman et al., 2016; Wang et al., 2017; Yang et al., 2017). To address these issues, NPs have attracted much attention due to their physicochemical properties, drug targeting efficiency, increased uptake, and bio-distribution (Eleraky et al., 2020; Karimi et al., 2016). Among NPs, polymeric NPs are the most used because they have several advantages. They protect drugs from degradation, increase their solubility, and promote controlled release and drug targeting (Kumari, Yadav & Yadav, 2010).

PLGA is one of the most preferred polymers for preparing polymeric NPs, and it has been approved by the FDA as a biocompatible, biodegradable polymer. It is also widely used for research in the pharmaceutical industry as a desired drug carrier (Abdollahi & Lotfipour, 2012; Kim et al., 2014). Thanks to their controlled release properties (Gaspar et al., 2018; Silva et al., 2014), PLGA NPs contribute to more effective antimicrobial properties of the active substance with no degradation. They also have the potential for oral administration (Hariharan et al., 2006; Mukerjee & Vishwanatha, 2009). There are several reports in the literature on the oral use of PLGA NPs loaded with antimicrobial agents (Abdollahi & Lotfipour, 2012). Antimicrobial agents such as rifampicin, isoniazid, pyrazinamide, and ethambutol used orally against *Mycobacterium tuberculosis* were encapsulated with PLGA, and their antibacterial activity was evaluated (Zhang et al., 2010). The results showed that PLGA NPs loaded with antimicrobial agents improved bioavailability and pharmacodynamics. In another study, the antibacterial activity of PLGA NPs loaded with azithromycin against *Salmonella typhi* was investigated. The results showed that azithromycin-loaded PLGA NPs were suitable for oral administration due to their favorable physicochemical properties and improved antimicrobial properties (Mohammadi et al., 2010).

The delivery of the antimicrobial agent to bacteria by NPs can occur *via* two mechanisms. In the first mechanism, the NP interacting with the cell wall or cell membrane carries the active substance into the target organism. The second mechanism is that the NPs adsorb to the cell wall and continue the release of antibacterial agents (Agarwal, Kumar & Rajeshkumar, 2017).

Plants have been used in traditional medicine in various cultures for many years. *O. acanthium* L. is an important herb used in medicine. This plant contains groups such as phenols, triterpenes, steroids (Garsiya et al., 2019), and biologically active compounds such as quercetin (Koc et al., 2015) and linoleic acid (Arfaoui et al., 2014). Due to its rich biologically active content of *O. acanthium* L., it is widely used in medicine.*O. acanthium* L. is used in traditional medicine as an anti-inflammatory, antitumor, and cardiotonic agent (Garsiya et al., 2019). Also, there is research in modern medicine on the properties of *O. acanthium*, such as bactericidal, cardiotonic, hypotensive, hemostatic, and antihypotonic (Khalilov et al., 2003; Tyumkina et al., 2009). In a study, the antibacterial properties of n-hexane and methanol extracts of *O. acanthium* seeds against Gram-positive bacteria (*S. aureus*, *S. epidermidis*, *M. loteus*) and Gram-negative bacteria (*E. coli* and *K. pneumonia*) were investigated by the MIC test (Zare et al., 2014). The methanol extract showed antibacterial activity against Gram-positive and Gram-negative bacteria. N-hexane showed no inhibitory activity against Gram-negative bacteria. In another study, the antibacterial activity of the leaf extract of *O. acanthium* was evaluated by MIC against *B. subtilis*, *X. euvesicatoria*, *L. plantarum*, and *A. fischeri* (Móricz et al., 2017). The results showed that the leaf extract had an antibacterial effect on the bacteria used.

In this study, OAE was encapsulated with PLGA and characterized. The average particle size, zeta potential, and polydispersity index values were determined using dynamic light scattering (DLS). The morphology of the OAE–PLGA NPs was demonstrated by SEM. The encapsulation efficiency, loading capacity, and *in vitro* release profile were determined using a UV-Vis spectrophotometer. The antibacterial effect of OAE–PLGA NPs against *S. aureus* was determined using the microdilution method. In addition, the antibacterial activity of the most abundant constituents of *O. acanthium* against *S. aureus* were investigated by molecular docking analysis to gain a better understanding of the mechanisms of action of these molecules. In addition, 50-ns MD simulation was performed to gain structural insight into the binding mode of the dynamic structure of the complex system. In the light of the information obtained from this study, it was revealed that quercetin in the OAE content interacted with the catalytic pocket binding residue, which is important for the inhibition of *S. aureus MurE*. Finally, the mutagenic and cytotoxic activity of free OAE and OAE-PLGA-NPs were evaluated by Ames test and MTT test.

## Materials and Methods

### Materials

PLGA, dichloromethane (DCM) and polyvinyl alcohol (PVA), citric acid monohydrate, sodium dihydrogen phosphate monohydrate, 4-nitro-*o*-phenylenediamine (NPD), magnesium sulfate heptahydrate, potassium phosphate, D-biotin, sodium chloride, magnesium chloride hexahydrate, disodium hydrogen phosphate dihydrate, potassium chloride, Nutrient Broth (No 2), L-histidine, Dulbecco’s Modified Eagle Medium (DMEM) and sodium ammonium phosphate tetrahydrate were purchased from Sigma–Aldrich (MO, USA). The *O. acanthium* extract (code: 5782ECH) was obtained from AZELIS TR KIMYA (Istanbul, Turkey). Agar was purchased from Difco (MD, USA). Muller-Hinton broth and Muller Hinton agar were purchased from Oxoid (Basingstoke, United Kingdom). Sodium azide was obtained from Merck (Darmstadt, Germany). HaCaT cell line was purchased from Thermo Fisher Scientific. 3-(4,5-Dimethylthiazol-2-yl)-2,5-Diphenyltetrazolium Bromide (MTT) was purchased from Biomatik. Fetal Bovine Serum (FBS) and Penicillin-Streptomycin (10X) Solution were purchased from Biological Industries.

### Methods

#### Fabrication of OAE–PLGA NPs

PLGA NPs loaded with OAE were prepared using a double emulsification technique (Budama-Kilinc, 2019b; Dewangan et al., 2022; Shabestarian et al., 2021). A total of 100 mg of PLGA was dissolved in 6 mL of DCM. Then, 10 mg of OAE was dissolved in 2 mL of water and added to 2 mL of PLGA. The emulsion (w/o) was formed by sonication with 70 W energy for 3 min. Then, 10 mg of PVA was dissolved in distilled water. The obtained w/o emulsion was added dropwise to the PVA solution. Then, the formation of double emulsions (w/o/w) was started with the homogenization process, that is, the sonication of the mixture for 5 min at 70 kW. Subsequently, OAE–PLGA NPs were washed through three centrifugation cycles at 11,200*g* for 35 min, discarding the supernatant and re-suspending the pelletized NPs in deionized water. The NPs were filtered through a filter, which is a cellulose membrane with a pore size of 0.45 µm, and lyophilized in order to perform the DLS and SEM analyses and antibacterial activity and genotoxicity tests.

#### Preparation of the OAE standard curve

For this study, the standard curve of the OAE was determined using a UV-Vis spectrophotometer. Seven stock solution concentrations (1.5625; 3.125; 6.25; 12.5; 25; 50; 100 μg/mL) were prepared for OAE, and UV absorbance of all concentrations was measured at 323.8 nm triplicate for each sample. The absorbance graph was plotted against the curve concentration, and the equation was obtained as y = 0.0033x (R^2 ^= 0.9992). The curve equation was used to determine both the encapsulation efficiency and the loading capacity (Ercin et al., 2022).

#### DLS analysis

The average particle size, polydispersity index (PdI), and zeta potential analyses of OAE–PLGA NPs were performed using a Zetasizer Nano ZS device (Malvern Instruments, Malvern, UK) equipped with a 4.0 mV He-Ne laser (633 nm) and operated at 25 °C.

#### FE-SEM analysis

The morphology of the OAE–PLGA NPs was demonstrated using FE-SEM (Apreo 2; Thermo Scientific, Waltham, MA, USA) (Adedokun et al., 2022). The sample containing OAE–PLGA NPs were dispersed in distilled water and placed in an ultrasonic bath for 45 min to sonicate. The sample was then prepared by dropping 10 µL of OAE–PLGA NPs onto the glass and drying at room temperature for 24 h. The sample was then placed in an ultrasonic bath. FE-SEM images were acquired using an in-lens detector at 100 kx magnification and 1.00 kV electron voltage.

#### Determination of encapsulation efficiency and loading capacity

The supernatant was taken after centrifugation of the OAE-loaded PLGA NPs to determine the encapsulation efficiency, and the amount of free OAE in the supernatant was calculated using an equation obtained from the OAE standard curve. The encapsulation efficiency was calculated using Eq. (1). The loading capacity for the OAE–PLGA NPs were calculated using Eq. (2).

(1) $$EE\;\%  = {{The\;total\;OAE - free\;OAE} \over {The\;total\;LEO}} \times 100$$

(2) $$LC\;\%  = {{The\;total\;OAE - free\;OAE} \over {Total\;Amount\;of\;the\;Nanoparticles\;Weight}} \times 100$$

#### *In* vitro release profile of OAE–PLGA NPs

The release of OAE from PLGA NPs was determined using the dialysis membrane method (Budama-Kilinc, 2019b; Kumari, Tyagi & Sangal, 2022). A total of 1 mg of OEA-PLGA NPs were suspended in 1 mL of distilled water and placed on pre-wetted dialysis membranes. The release was carried out in 100 mL of PBS (pH 7.4) medium in a shaking water bath maintained at 37 °C at 120 rpm. At fixed time intervals, the sample was taken from 1 mL of the release medium and added with an equal volume of buffer to keep the volume of the release medium constant. OAE amount in the release medium was analyzed using a UV-Vis spectrophotometer. The amount of OAE was calculated according to Eq. (3).

(3) $$Release\;\;\%  = {{Released\;Amount\;of\;\;OAE} \over {Total\;Amount\;of\;OAE}} \times \;100$$

#### Antibacterial activity

The antibacterial activity of free OAE and OAE-PLGA NPs on *S. aureus* ATCC 25923 was evaluated by MIC assay. Bacterial culture was activated on Mueller–Hinton agar at 37 °C for 24 h. After then, three colonies were transferred to a fresh medium (MHB) to culture the bacteria and grown overnight at 37 °C. The fresh bacterial culture was set to OD_600_ = 0.01 (5 × 10^6^ cfu/mL). Free OAE and OAE-PLGA NPs were dissolved in distilled water, and serially diluted in MHB medium in 96-well plates to a final volume of 100 µL per well. Then, 5 µL of bacterial inoculum was added to each well. OAE-PLGA NP concentration was used in the range of 0.125 to 1 mg/mL, and free OAE concentration in the range of 0.093 to 0.75 mg/mL (the amount of OAE was calculated based on the amount loaded on the OAE-PLGA NPs). The experiment was performed in three technical replicates for each sample. The microplates were incubated at 37 °C for 24 h. The plates were analyzed at 540 nm with an ELISA reader (Multiskan GO Microplate Spectrophotometer; Thermo Scientific, Waltham, MA, USA) (Khoshkhounejad et al., 2021). Percentage inhibition of bacterial growth (%) was determined according to Eq. (4); A_c_ is the absorbance value of the negative control and A_t_ is the absorbance value of the samples.

(4) $$Inhibition\;\left( {\;\% } \right) = {{{A_c} - {A_t}} \over {{A_c}}} \times 100$$

#### Molecular docking and MD analysis

The antibacterial efficacy of *O. acanthium*, which has anticancer, antioxidant, anti-inflammatory, analgesic, antipyretic, hypotensive, antiepileptic, wound-healing, and ACE inhibitory effects, was evaluated for the first time using molecular docking analysis. Antibacterial activity of OAE against *S. aureus*. was investigated at the molecular level.

The main structures formed by *O. acanthium* are flavonoids (such as apigenin, quercetin, and luteolin), phenylpropanoids, lignans, triterpenoids, sesquiterpene lactones, sterols, and three active antibacterial compounds, such as linoleic acid, linolenic acid, and germacranolide sesquiterpene lactone (Al-Snafi, 2020). Quercetin has been found in the aerial parts of the plant, the leaves, and the flowers. However, quercetin glycosides have also been isolated from herbs. In various extracts, such as ethanol, methanol, and acetone, the flowers contain 30 to 40 mg/L of quercetin. The leaves also contained 40 to 85 mg/L of quercetin. The main fatty acids in *O. acanthium* are linoleic acid (65.9%), oleic acid (18.8%), palmitic acid (5.8%), stearic acid (2.6%), and pentadecanoic acid (Garsiya et al., 2019; Karl, Mueller & Pedersen, 1976; Koc et al., 2015). The most abundant constituent of *O. acanthium* are quercetin and linoleic acid were preferred as the active ligands, and the protein data bank provided the crystal structure of *S. aureus* (PDB ID: 4C13) (Ruane et al., 2013) as the active receptor for performing molecular docking analysis. The Glide SP module of Maestro version 11.4 from Schrodinger Software (Friesner et al., 2004; Halgren et al., 2004) was implemented for molecular docking analysis and ADME calculations. The ChEBI service (Hastings et al., 2016) was used to generate the possible 3D molecular geometries of ligands. Both ligands that were considered possible inhibitors of *S. aureus* were transferred to the builder panel, and then the optimization process was performed using the LigPrep module. For energy minimization, the OPLS3 force field was used (Harder et al., 2016). For the docking analysis, the obtained geometries of the inhibitory ligands with the conformation with the lowest energy were used. The 3D crystal structure of *S. aureus* MurE, which was obtained at a resolution of 1.9 and formed a single A-chain of 501 amino acids containing natural ligands, phosphate, potassium, chloride, and magnesium ions, was extracted from the protein database to perform the molecular docking analysis. Magnesium ions that were near the natural ligand were retained, while all water and ions that were not in the binding region of the natural ligand were deleted, as in the literature (Azam, Saha & Jupudi, 2019). Polar hydrogens were added, binding orders were assigned, and preprocessing was performed. Defective in the receptor structure were analyzed and loaded with PROPKA (Olsson et al., 2011) at pH 7.0. The receptor structure was optimized and minimized (Sastry et al., 2013) with the protein preparation tool using the OPLS3 force field. This process was performed, and it was expected that the root mean square deviation (RMSD) of the heavy atoms would converge to 0.30. By creating a 3D grid box centered on the center of gravity of each ligand, all residues containing thiol and hydroxyl groups were identified in the binding region of the receptor, and each ligand was docked to the receptor. Also, all pharmacokinetic and physicochemical properties of the different ligands binding to the same receptor were calculated using the Qik-Prop module (Nija, Rasheed & Kottaimuthu, 2020). The pharmacokinetic potential of both ligands thought to have inhibitory properties against *S. aureus*, such as molecular weight (M_W_), percentage of oral absorption by humans, estimated octanol/water partition coefficient (QPlogPo/w), polar surface area (PSA), and Lipinski’s Five Rules compatibility properties, were also obtained. Furthermore, to investigate effect of the most abundant constituent of *O. acanthium* on *S.aureus* by dynamic system and validate the stability of the complex, MD simulation (~13,820 water molecules and 49,119 atoms) was performed for 50 ns, and the DESMOND (Bowers et al., 2006; Schrödinger, 2021) module of the same program was used.

#### Ames/Salmonella assay

The Ames test was performed using the standard plate incorporation method (Maron & Ames, 1983; Ying et al., 2022; Zhang et al., 2022). TA98 and TA100 strains of *S. typhimurium* were used in the study.

After checking the genotypic characteristics of the test strains, including the histidine requirement, rfa mutation, plasmid pKM101, and uvr B mutation, the experiment was started (Akin et al., 2016). In the experiment, 0.25, 0.5, and 1 mg/plate concentrations of OAE-PLGA NPs were used, while free OAE concentrations were determined according to the loading capacity.

Briefly, 0.1 mL of the bacterial culture (1–2 × 10^9^) and the test sample were added to the top agar and mixed with a vortex. This mixture was then poured onto the surface of a minimal glucose agar (MGA) plate. The plates were incubated at 37 °C for 48 h. After incubating the plates, mutagenicity was assessed by comparing the number of revertant colonies formed by the tested samples with the number of revertant colonies formed on the negative plates. Experiments were performed in triplicate for each sample.

#### MTT assay

Cytotoxicity of free OAE and OAE–PLGA NPs were determined by MTT test. Briefly, L929 cells were seeded in 96-well plates (1 × 10^4^ cells/well) and OAE and OAE–PLGA NPs were applied after 24 h of culture. OAE–PLGA NPs concentrations were determined as 0.125, 0.25, 0.5, 0.75, 1 and 2, mg/mL, while concentrations for free OAE was applied according to the loading efficiency. After treatment, cells were cultured for 24 h in a 37 °C humidified 5% CO_2_ incubator. After 24 h, 5 mg/mL MTT was added to each well, and cells were incubated for 4 h in a 37 °C 5% CO_2_ incubator. Then the supernatant was removed and 100 µL of DMSO was added. The plate was measured using an ELISA reader (EPOCH; Biotek, Winooski, VT, USA) at 570 nm. Mean optical density (OD) values were used to estimate cell viability. Cell viability was calculated using Eq. (5).

(5) $${\rm{Cell}}\;{\rm{viability}}\;\;\%  = {{{\rm{OD}}\;{\rm{of}}\;{\rm{Experimental}}\;{\rm{Group}}} \over {{\rm{OD}}\;{\rm{of}}\;{\rm{Control}}\;{\rm{Group}}}} \times 100\;$$

#### Statistical analysis

Statistical analysis of the mutagenicity and cytotoxicity study was performed using one-way analysis of variance (ANOVA) to compare values between control and treated groups. Values showing *p* < 0.05 were considered statistically significant.

## Results and discussion

### Characterization results of OAE-PLGA-NPs

#### DLS analysis

The hydrodynamic size, distribution, and surface charge of NPs were determined using DLS, the most widely used method for NP characterization (Budama-Kilinc, 2019a; Egil et al., 2020; Nemati et al., 2022; Samling et al., 2022). In this study, the average size, polydispersity index (PdI), and zeta potential of OAE–PLGA NPs were measured using DLS principles.

**Figure 1:**
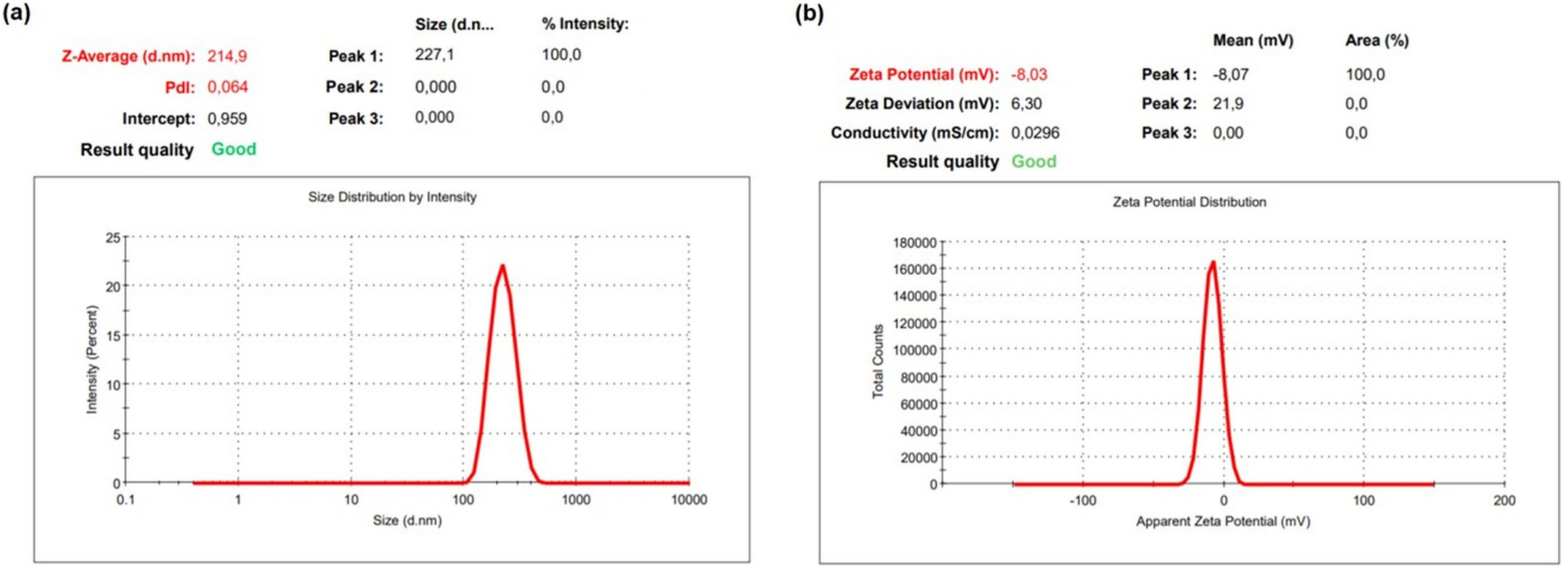
DLS analysis. DLS analysis of OAE–PLGA NPs: (A) average particle size graph, (B) zeta potential graph.

The DLS result of OAE–PLGA NPs was shown in Fig. 1. It was found that the average particle size was 214.9 ± 6.77 nm and the zeta potential was −8.03 ± 0.85 mV. The OAE–PLGA NPs exhibited a narrow size distribution with PdI values of 0.064 ± 0.013.

NPs are particles with a size of less than 100 nm (Borm et al., 2006; Dowling, 2004). However, the size of polymeric NPs ranges from 10 to 1,000 nm, and they are used in various applications (Gheffar et al., 2021; Hamzaoui & Laraba-Djebari, 2021; Ni et al., 2021; Roberts et al., 2020). The double emulsion method allows the preparation of NPs with a size larger than 100 nm in the synthesis of polymeric NPs (Dorjsuren et al., 2020; Pieper & Langer, 2017; Sousa et al., 2017). The size of OAE–PLGA NPs synthesized by the double emulsion method was compatible with the particle sizes synthesized in previous studies.

The PdI value is a measure of the homogeneity of NP size. A PdI value close to zero indicates a homogeneous distribution, while a PdI value close to one indicates a completely heterogeneous and polydisperse particle population (Al-Mahallawi, Abdelbary & Aburahma, 2015; Gebreel et al., 2021). In our study, it was found that the PdI values of the synthesized OAE–PLGA NPs were smaller than 0.1. This result indicates that the OAE–PLGA NPs have good homogeneity and uniform particle size distribution.

The zeta potential is the total charge acquired by particles in a given medium. This charge value is an indication of the potential physical stability of NP distribution (Dhas, Ige & Kudarha, 2015). The electric charge of the OAE–PLGA NPs were negative due to the terminal carboxyl groups in PLGA (Bacanli et al., 2021; Budama-Kilinc, 2019b; Zhang et al., 2021).

#### Encapsulation efficiency and loading capacity

Encapsulation is a strategic method to keep drug molecules stable and increase their efficacy. Therefore, encapsulation efficiency and loading capacity are essential calculations and measurements for NP preparation (Shen et al., 2017). Standard curve equation of OAE was y = 0.0033x (R^2^ = 0.9995). The encapsulation efficiency was calculated to be 91% using Eq. (1), and the loading capacity was determined to be 75.83% using Eq. (2). The results of encapsulation efficiency and loading capacity show that OAE was encapsulated and that OAE–PLGA NPs were successfully obtained.

#### *In* vitro release kinetics of OAE–PLGA NPs

**Figure 2:**
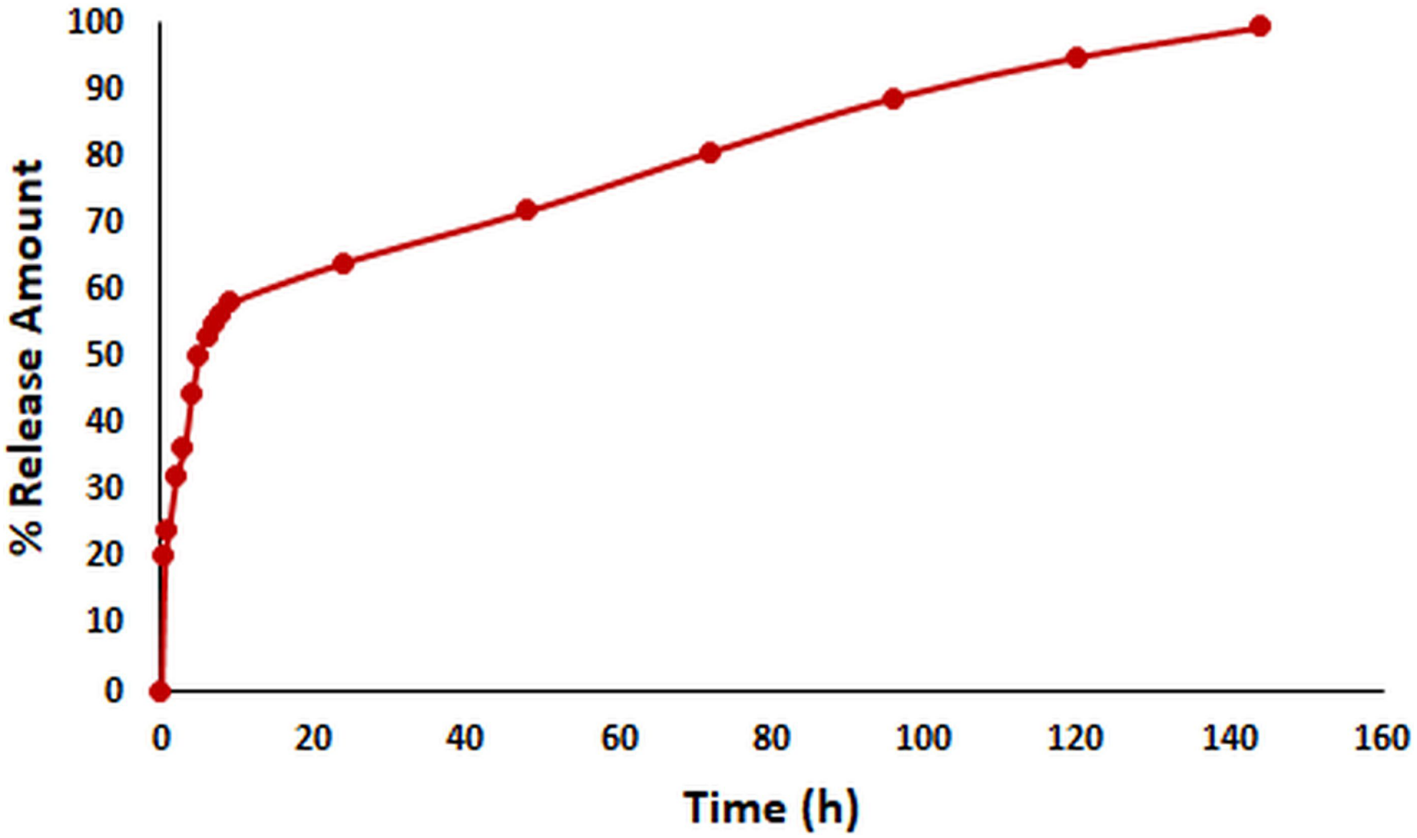
*In vitro* release study result. *In vitro* release profile of OAE-PLGA NPs.

*In vitro* release kinetics are crucial as an indicator of the pharmacokinetic and pharmacological effects of a drug *in vivo* (Abdelkader et al., 2020). The *in vitro* release kinetics of OAE–PLGA NPs were performed in PBS buffer (pH = 7.4) using the dialysis membrane method (Folle et al., 2021) and monitored for 144 h. The percentage of OAE released as a function of time was given in Fig. 2. The results showed that 58.18% of OAE was released within 9 h, 64.24% within 24 h, and 99.39% within 144 h. The *in vitro* release profile of the OAE–PLGA NPs showed a biphasic release pattern. The initial rapid release may be attributed to the rapid release of OAE entrapped near the surface of the NPs. However, the sustained release could be related to the OAE entrapped deep in the core of the PLGA NPs (Chourasiya, Bohrey & Pandey, 2016; Radwan et al., 2021).

#### FE-SEM analysis

**Figure 3:**
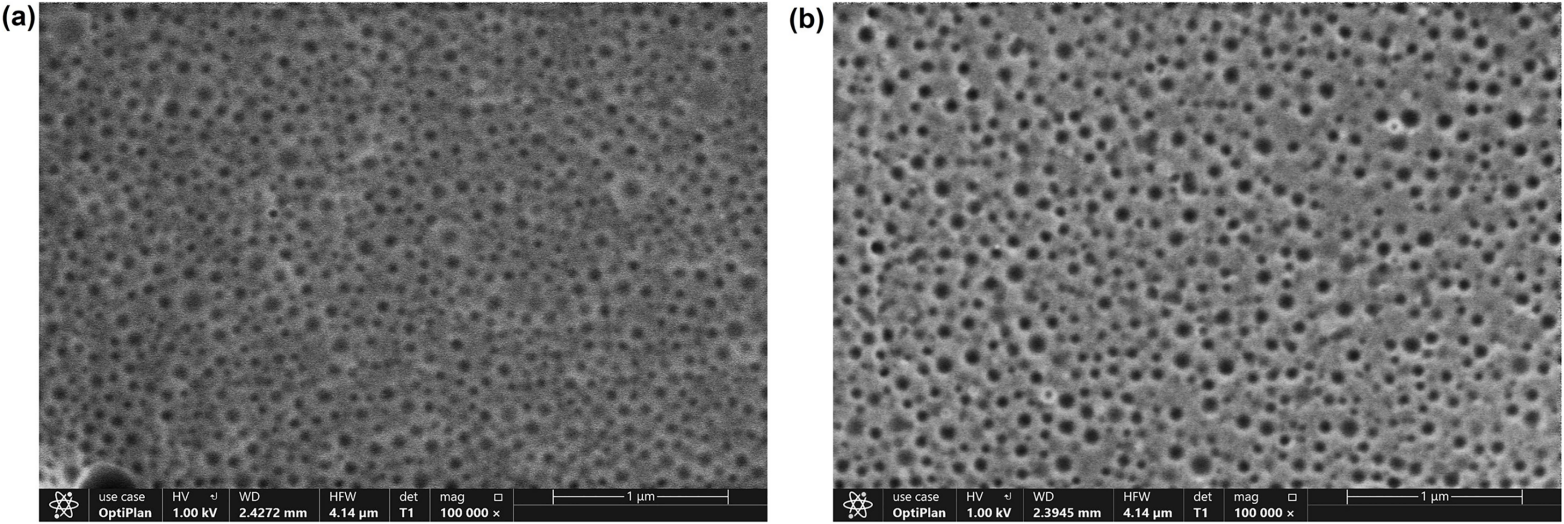
FE-SEM analysis. FE-SEM image of blank PLGA NPs (A), OAE-PLGA NPs (B).

The morphology of the OAE–PLGA NPs and blank PLGA NPs were observed using FE-SEM (Fig. 3). As shown in the figure, both NPs (Figs. 3A and 3B) were spherical morphology with a homogeneous distribution were produced (Adedokun et al., 2022; Malewicz et al., 2022).

### Antibacterial activity assay

The antibacterial activity of free OAE and OAE–PLGA NPs were examined using the broth microdilution method. The bacterial growth inhibition of free OAE and OAE–PLGA NPs on *S. aureus* was 99% and 69%, respectively (Table 1).

**Table 1:** Antibacterial activity assay results. The MIC and inhibition values of OAE and OAE–PLGA NPs.

Treatment	Concentration	% Inhibition ± SD	MIC (mg/mL)
Free OAE	0.75	99.0 ± 0.032	0.75
	0.37	93.4 ± 0.016	
	0.18	68.5 ± 0.034	
	0.093	45.6 ± 0.065	
OAE-PLGA-NPs	1	69.0 ± 0.001	>1
	0.5	67.7 ± 0.007	
	0.25	65.4 ± 0.013	
	0.125	59.4 ± 0.007	

Radwan et al. (2021) reported that the encapsulation of the drug with PLGA NPs resulted in a significantly slower and more controlled drug release compared with the free drug. This explains the lower antibacterial activity compared to free OAE, considering the amount of OAE released from the NPs in the first 24 h (64%). Our results are compatible with the literature (Chaudhary & Kumar, 2014; Hill, Taylor & Gomes, 2013; Mohammadi et al., 2010; Simon et al., 2020). 

OAE contains active compounds such as quercetin (Koc et al., 2015) and linoleic acid (Matthaus, Ozcan & Al-Juhaimi, 2014). Many studies in the literature have reported the antibacterial activity of quercetin (Kost et al., 2020; Shu et al., 2011) and linoleic acid (Bentrad, Gaceb-Terrak & Rahmania, 2017; Jummes et al., 2020; Kusumah et al., 2020). The antibacterial activity shown by OAE could be due to these active compounds. The antibacterial mechanism of quercetin can be used in several ways. These mechanisms include alteration of cell permeability, harm to the bacterial cell wall, and inhibition of nucleic acid synthesis, which may lead to altered protein synthesis and decreased enzyme activities (Wang et al., 2018). The target of the antibacterial mechanism of linoleic acid is the cell membrane. Linoleic acid dissolves the cell membrane and allows cell metabolites to leak out and the cells to break down (Desbois & Smith, 2010).

### Molecular docking and MD analysis results

**Figure 4:**
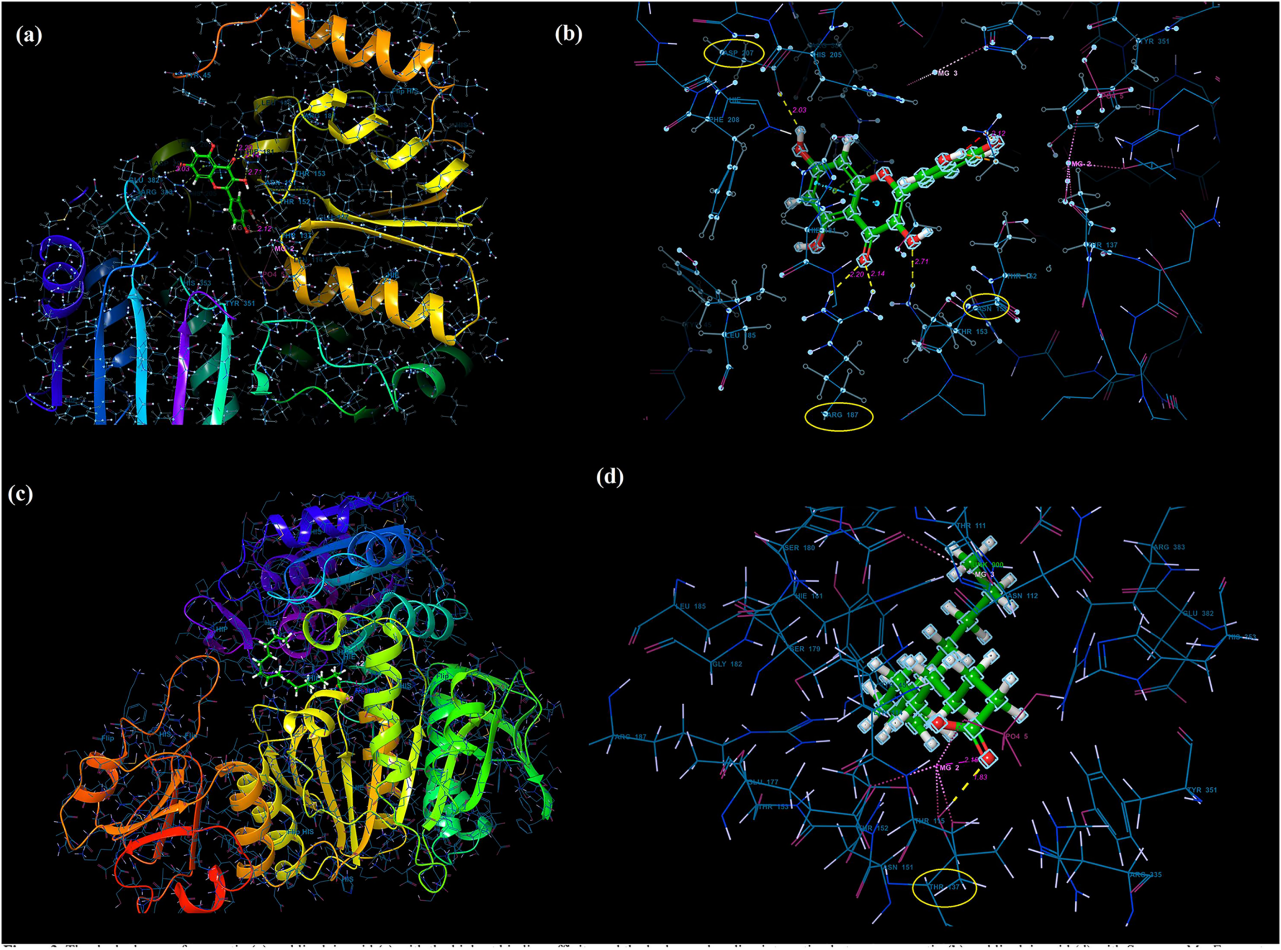
Molecular docking results. Docked pose of quercetin (A) and linoleic acid (C) with the highest binding affinity and hydrogen bonding interactions between quercetin (B) and linoleic acid (D) with the *S. aureus* MurE receptor.

**Figure 5:**
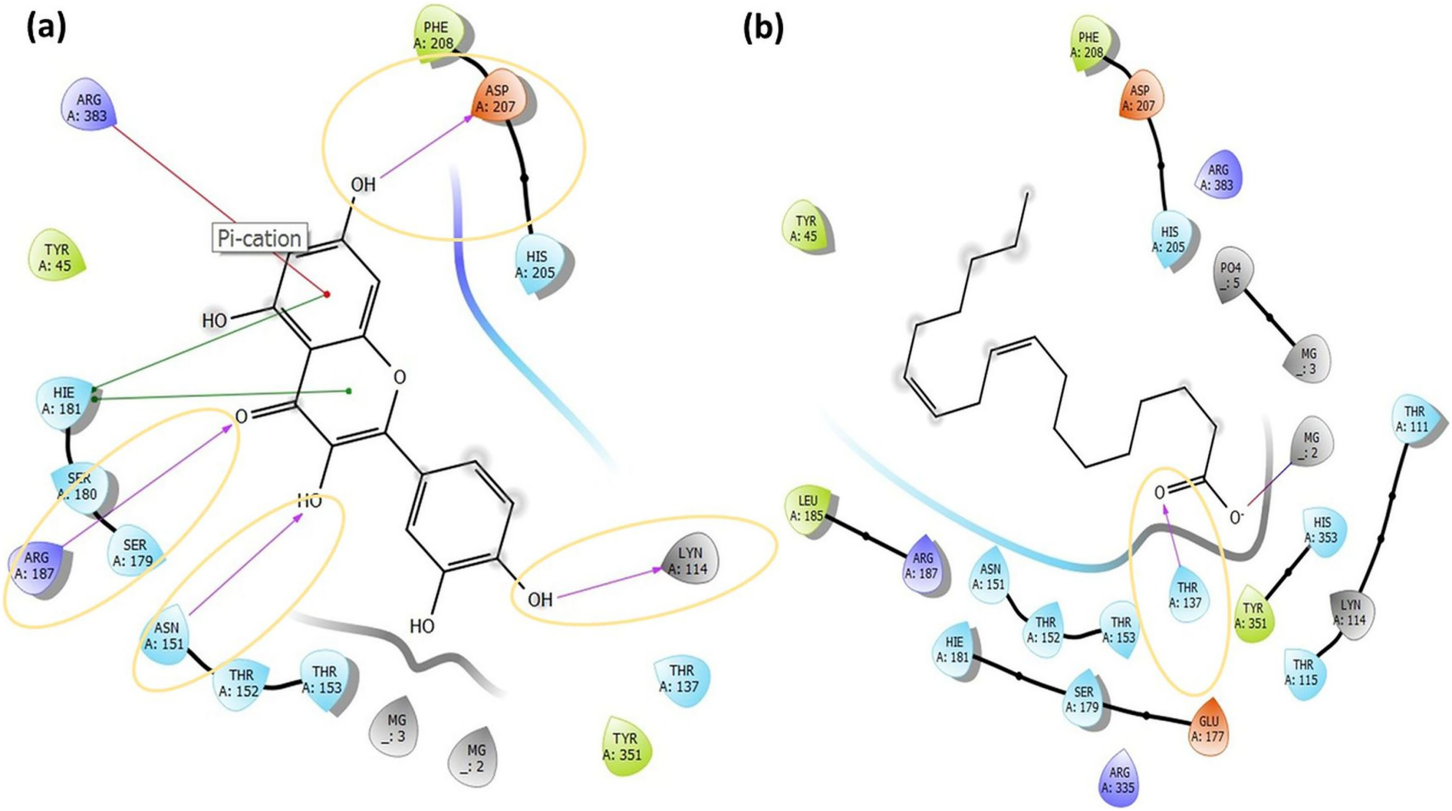
The active binding sites and the 2D ligand interactions. The 2D binding interactions with quercetin (A) and linoleic acid (B) major compounds in *O. acanthium* with the active binding site of *S. aureus* MurE.

Molecular docking analysis was performed to model the possible binding conformations of active compounds, such as quercetin and linoleic acid in *O. acanthium* against *S. aureus*. Based on the molecular docking analysis, the binding affinity to the target receptor and the potential of the binding compounds to become drugs can be predicted. While binding affinity is expressed by the value of the docking score, lower values for binding affinity mean that a compound requires less energy to bind, *i.e*., its potential to bind to the target receptor is higher (Baker et al., 2007; Tassa et al., 2010). The binding affinities were determined and compared for quercetin and linoleic acid in *O. acanthium* against *S. aureus*. in Table S1. The binding conformation of the two ligands, such as quercetin (Fig. 4A) and linoleic acid (Fig. 4C), with the highest binding affinity, and the hydrogen bonding interactions between quercetin (Fig. 4B) and linoleic acid (Fig. 4D) against the *S. aureus* MurE receptor were shown in Fig. 4. The more negative the docking score, the stronger the binding affinity of the ligand to the receptor. In inhibiting *S. aureus*, quercetin had the lowest docking score of −6.770 kcal/mol. Quercetin is known as penta hydroxy flavone a form the flavonoid group and has five hydroxyl groups in its structure. Because of the five hydroxyl groups, it was very well bound to the active binding site of *S. aureus* MurE. Linoleic acid is known as cis, cis-9,12-octadecadienoic acid and has only one binding carboxyl group. *S. aureus* is an important human pathogen and is among the leading causes of skin and soft tissue and device-related infections, as well as infective endocarditis. The *S. aureus* MurE enzyme is one of the potential targets for the development of new therapeutic agents due to its high substrate specificity and ubiquitous nature among bacteria (Azam, Saha & Jupudi, 2019). The residues and binding types where both ligands bind to the active binding site of the *S. aureus* receptor are also shown in Fig. 5.

The higher affinity value of quercetin is due to the fact that the OH and O atoms of quercetin, as shown in Fig. 5A, interact harmoniously with the corresponding residues at the active binding site of the receptor. Three hydroxyl groups and one oxygen atom in quercetin were connected to the residues (ASP207, ASN151, LYS114, and ARG187) at the active binding site *via* hydrogen bonds (2.03, 2.71, 2.12, 2.14 and 2.20 Å), as shown in Table S1. In a study on the *Staphylococcus aureus* MurE inhibitor, it was observed that the molecule whose inhibitory activity was investigated provides this activity by making hydrogen bonds with the active binding site of the similar residues with ASN151, THR152, SER180, ARG187 AND LYS219 (Azam, Saha & Jupudi, 2019). These hydrogen bonding interactions are considered important interactions for the stabilization of the inhibitor within the catalytic pocket of *S. aureus* MurE.

Quercetin made also strong hydrogen bonds with the similar residues ASN151 and ARG187, which are available in domain 2 region covering from 99 to 332 residues to the active binding site of *Staphylococcus aureus* MurE in Fig. 5A. The powerful interactions that supported stronger binding were also two pi-pi stacking interactions between the benzene rings of quercetin and the HIE181 residue and a pi-cation interaction between the benzene ring with the ARG383 residue. Non-covalent interactions between the aromatic rings of quercetin and HIE181, as well as an electron-rich pi system and an adjacent cation, such as ARG383, contributed to the stronger binding seen in Fig. 5A. Apart from the noncovalent interaction from ARG383 residue at domain 3 which consists of residues from 333 to 493, hydrogen bonding interactions in the binding pocket of domain two appeared to play a important role for the stabilization of the inhibitor.

Linoleic acid was also bound to the active binding site with an energy of −2.734 kcal/mol. Figure 5B built a hydrogen bond interaction (1.83 Å) between the oxygen atom and the THR137 residue and a salt bridge (2.16 Å) interaction between the oxygen atom of the carboxyl group and the magnesium ion.

Figures S1A and S1B shows the molecular electrostatic potential surface of the binding pocket of the *S. aureus* MurE receptor and ligands for quercetin (Fig. S1C) and linoleic acid (Fig. S1D), respectively. Considering the difference between the binding affinity energy values and the extent of interaction at the active binding site, quercetin was found to have more effective binding than linoleic acid, so that quercetin has a stronger inhibitory effect on *S. aureus* than linoleic acid. *In silico* molecular docking analysis results show that *O. acanthium* extract is promising as a drug candidate with strong antibacterial activity against *S. aureus*, due to the quercetin compound, whose antibacterial activity has also been proven in the literature and presented in this study. The ADME properties, which determine the kinetics of drug exposure in tissues and establish the performance and pharmacological activities of the active ingredients as drugs, were calculated for quercetin and linoleic acid in *O. acanthium*, and the results were shown in Table S2. Quercetin and linoleic acid both have a low molecular weight. While quercetin has four donors and five acceptors, linoleic acid contains only one donor and two acceptors. The calculated Caco-2 and MDCK permeability values for linoleic acid were in the medium range, but for quercetin, they were poor.

**Figure 6:**
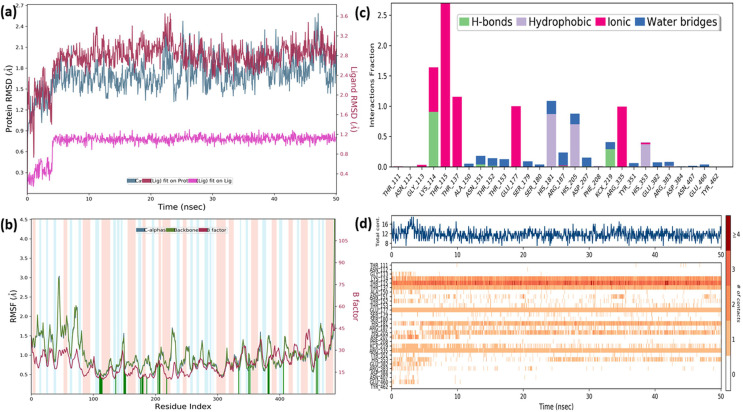
MD results. RMSD profile of Cα (A), RMSF profile of 4C13 residues (B), interactions fraction diagram of quercetin (C), interaction counts profile of quercetin with different residues of 4C13 (D), during 50 ns MD.

In order to gain insight into the inhibitor mechanism by dynamic interaction of OAE’s active compound, such as quercetin, on *S. aureus* MurE receptor and its stability, RMSD and RMSF were analyzed relative to the initial structure by subjecting to the 50-ns simulations. Figure 6A presents the *S. aureus* MurE receptor backbone RMSDs for Cα with green and ligand RMSD with pink.

According to the MD analysis, during the first 5 ns step, calculated RMSD values of the protein backbone Cα indicated updraft from 1.2 and to 1.8 Å and remained stable around 2.1 Å for the remaining simulation time. At the same time, it was seen that the calculated ligand RMSD value increased from 0.3 to 0.8 Å under 10 ns and remained stable around 0.8 Å for the next 40 ns. This balance RMSD value indicated that the entire complex is in equilibrium. In Fig. 6B, the peaks are expressed by the protein domains that fluctuate the most during simulation, while the residues interacting with the inhibitory structure are also represented by green vertical lines. For the backbone, these fluctuation values seem to increase from 1.5 to 3.0 Å for the first 100 residue indexes. The backbone and Cα value of the catalytic pocket residues (99–332 residues) bound to the inhibitor exhibited root mean square fluctuation (RMSF) values are in the range of 0.55–1.45 Å, respectively.

Trajectory analysis from the MD simulation revealed hydrogen bonding, hydrophobic, and ionic interactions of the inhibitor with the key binding residues of the catalytic pocket, as seen in Fig. 6C. Consistent with the results of the docking analysis, hydrogen bonds were observed with ASN151, THR152, LYS114, ARG383 and LYS219 which formylated at KCX219 in Figs. 6C and 6D, while ionic interactions were observed with GLY113, LYS114, THR115, THR137, GLU177 and ARG335. In complex structure, water-bridged interactions were observed between amino acid regions between ALA150, ASN151, THR152, SER179, ARG187, ASP207, TYR351, GLU382, ARG383 and GLU460, while hydrophobic interactions occurred in HIS181, HIS205 and HIS353 regions. The interaction counts and times of the inhibitor with the relevant residues are also seen in Fig. 6D. Interactions with THR115, LYS114, THR137, GLU177, ARG335 are expressed as interactions that exist during the 50 ns MD period.

**Figure 7:**
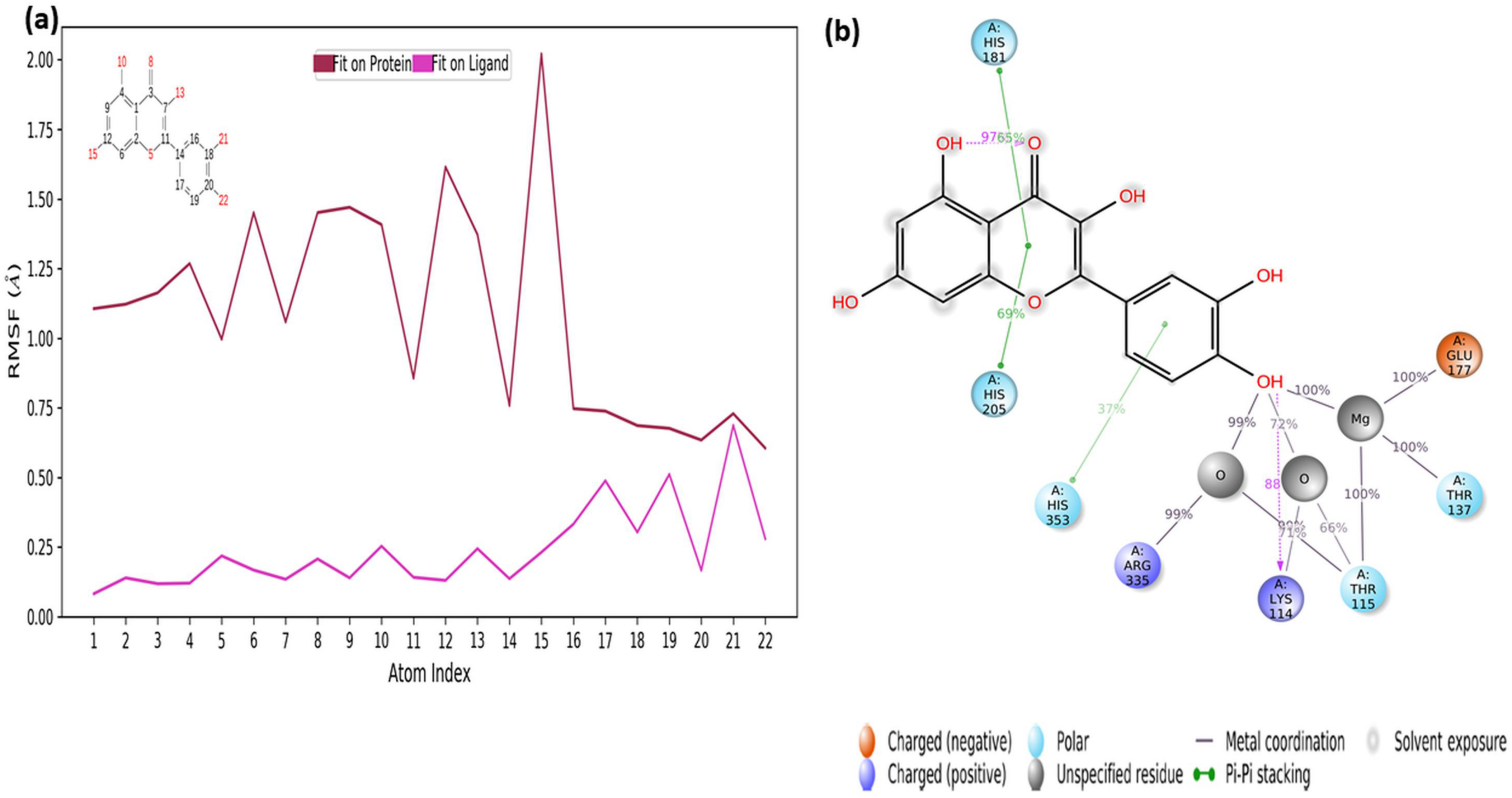
RMSF profile of quercetin. RMSF profile of atom of quercetin (A) interactions diagram of quercetin (B) with different residues of 4C13, during 50 ns MD.

The RMSF values of the atoms of the inhibitor are also given in Fig. 7A. Accordingly, the atoms that act together with the protein in the Ligand are given with their numbers. The hydroxyl group, defined by atomic number 22, was in water-mediated ionic interactions with ARG335, THR115, and THR137 (99%, 72%, and 100% of the MD trajectory, respectively). The hydroxyl oxygen of the same moiety formed a strong hydrogen bond of 88% of the MD trajectory with LYS114. Again, the same hydroxyl group made ionic interaction with GLU177 *via* the magnesium cation at 100% of the MD trajectory. In addition, pi-pi interactions with HIS181, HIS205 and HIS353 occurred of 65%, 69% and 37% of MD trajectory, respectively seen in Fig. 7B.

It is evident from above results that the strong hydrogen bonding interactions with LYS114 and LYS219 in Figs. 6C and 7B, and other hydrogen binding with ASN151 and THR152 in Fig. 6C, and also ionic interactions with THR115, THR137, GLU177 and ARG 335 and pi-pi interactions with HIS181, HIS205 and HIS353 are crucial for enhancing the activity value.

### Ames/Salmonella assay

**Figure 8:**
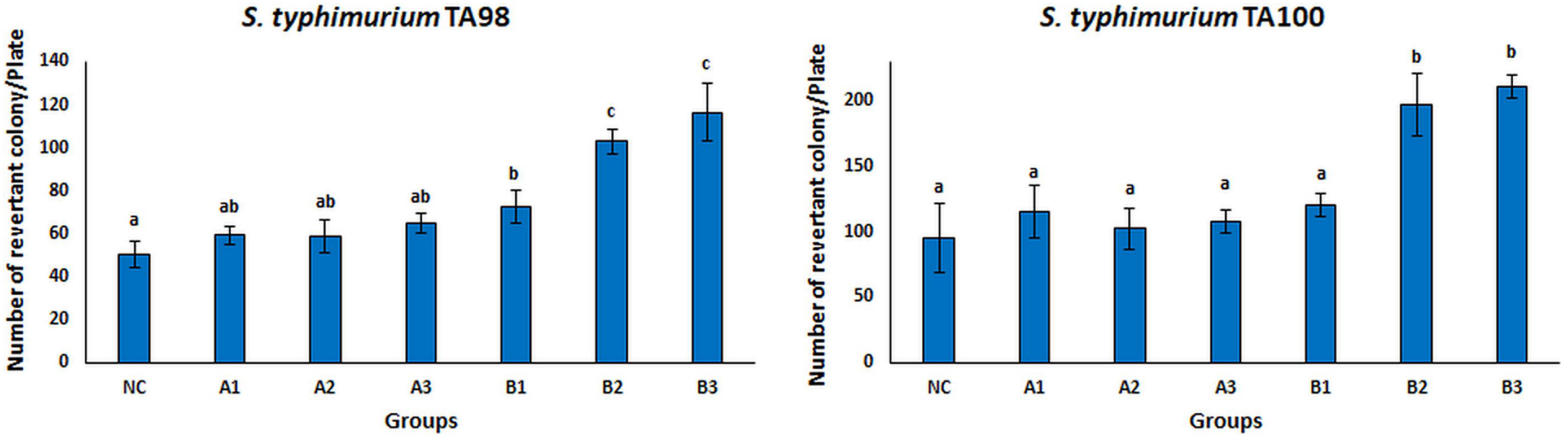
Mutagenicity results. Mutagenicity results of free OAE and OAE-PLGA NPs. NC, Negative control; A1, 0.25 mg/Plate of OAE-PLGA NPs; A2, 0.5 mg/Plate of OAE-PLGA NPs; A3, 1 mg/Plate of OAE-PLGA NPs; B1, 0.17 mg/plate of free OAE; B2, 0.38 mg/plate of free OAE; B3, 0.75 mg/plate of free OAE.

The Ames test is a real-time, sensitive, short-time test that represents the mutagenicity of chemical substances (Abdul Majid et al., 2022; Coppi et al., 2022; Tsai et al., 2020; Zhao et al., 2020). This test system is frequently used to investigate the mutagenicity of many chemical substances. There are different tests to measure whether mutation or genetic damage is present in microbial and mammalian cells, but the Ames test still plays an important role in testing chemicals for commercial use (Zeiger, 2019). Many strains of *S. typhimurium* (TA100, TA98, TA97, TA102, TA1535, TA 1537, and TA1538) were used for the Ames test. There are different mutations in the genes of these strains. *S. typhimurium* strains TA98 and TA100 are the most standardized strains against frameshift and base pair change mutations (Mortelmans & Zeiger, 2000). Therefore, the *S. typhimurium* TA98 and TA100 strains were used in this study. The mutagenicity results of free OAE and OAE–PLGA NPs were shown in Fig. 8. The results showed that the applied concentrations of OAE–PLGA NPs were not mutagenic (*p* > 0.05). However, 0.75 and 0.37 mg/mL concentrations of free OAE were found to cause both frameshift mutation (TA98) and base-pair substitution (TA100) (*p* < 0.05).

The fact that free OAE causes a mutagenic effect (*p* < 0.05) and that OAE–PLGA NPs have no mutagenic effect could be explained by the controlled release system. The controlled release system provides a much slower drug release. This eliminates the mutagenic effect. Many studies in the literature report that the toxicity of the drug is eliminated thanks to the controlled release system (Sousa et al., 2017; Zhang et al., 2021). The absence of any mutagenic effect of OAE–PLGA NPs tested on mutant strains of TA 98 and TA 100, two bacterial species, confirms the safety of these particles for use in diseases caused by *S. aureus* bacteria (Ballesteros-Ramírez, Durán & Fiorentino, 2021; Khan et al., 2021).

### MTT assay

**Figure 9:**
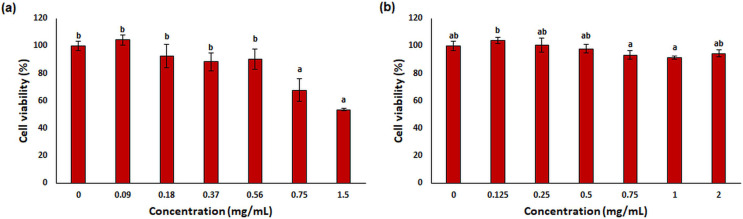
The cytotoxicity results. The cytotoxicity results (A) free OAE and (B) OAE-PLGA NPs. Different letters mean significant differences between the sample and control.

One of the most used tools in toxicity studies associated with nanoparticle-based therapies is cell cultures. They are simple, cost-effective, and do not pose any ethical problems. In addition, *in vitro* cell tests allow checking of the cellular environment and homogeneity, both morphologically and compositionally. This provides a deeper understanding of the biological and biochemical processes that occur during treatments (Razura-Carmona et al., 2022). L929 mouse fibroblast was used for *in vitro* cytotoxicity testing in this study. This cell line is often preferred by researchers because it is easy to control cell culture conditions (ISO, 2009) and responds more sensitively than primary cells (Nabavizadeh et al., 2022; Sharma et al., 2022). The results of cytotoxicity analysis on L929 fibroblast treated with free OAE and OAE-PLGA-NPs were given in Fig. 9. OAE-PLGA-NPs did not show cytotoxicity against fibroblast cells even at the highest concentration of 2 mg/mL (*p* > 0.05). However, 0.75 and 1.5 mg/mL concentrations of free OAE were cytotoxic to fibroblast cells (*p* < 0.05).

Paszel-Jaworska, Romaniuk & Rybczynska (2014) *Ziziphora clinopdioides* Lam. theoretically attributed the toxic effect of ethanolic extract (EEZC) to the chemical content of the extract. OAE’s toxicity may be due to its chemical content. Omokhua et al. (2018) reported that the antibacterial and antifungal activity of *Tithonia diversifolia* belonging to the Asteraceae family may be due to the toxicity of the plant extract. This may explain the antibacterial effect of *O. acanthium* extract (OAE) belonging to the Asteraceae family on *S. aureus*.

In our study, the toxicity of free OAE and OAE-PLGA-NPs were evaluated with two different tests. While OAE-PLGA-NPs did not cause toxicity in both test systems (*p* > 0.05), high concentrations of free OAE caused toxicity (*p* < 0.05). However, the concentration of 0.37 mg/mL was toxic (*p* < 0.05) in the Ames/Salmonella test, while it was non-toxic in the MTT test (*p* > 0.05).

Taherkhani (2015) reported that essential oil components have a very different mode of action in bacteria and eukaryotic cells. While they have potent bactericidal properties for bacterial cells, they alter apoptosis and differentiation in eukaryotes, interfere with post-translational modification of cellular proteins, and induce or inhibit some hepatic detoxifying enzymes. Therefore, they emphasized that essential oils could cause very different effects in prokaryotes and eukaryotes. OAE contains various components (Al-Snafi, 2020). These components may have caused different effects on prokaryotic and eukaryotic cells, such as essential oils.

## Conclusion

*S. aureus*, a human commensal microbe, has caused infections throughout history and is likely to continue to be a significant cause of human infections. The ability of *S. aureus* to rapidly develop antibiotic resistance provides an orientation towards alternative treatment methods for severe *S. aureus* disease. The resistance of *S. aureus* to existing antibiotics is both a serious health threat and an economic burden. Therefore, new antibacterial agents and innovative systems, such as nano-sized formulations with controlled release features, are urgently needed.

In literature studies, it has been found that delivery systems based on nanomaterials as drug carriers show great potentials in antibacterial therapy. Functional nanomaterials with antibacterial properties do not induce bacterial resistance and can suppress bacterial resistance by bypassing drug-resistant mechanisms while protecting important structural components of loaded antibiotics. Nanoparticle formulations are often preferred because of their properties that protect the antibacterial agent and increase its biocompatibility. In addition, nanoformulations can increase drug-induced antibacterial activity by promoting interaction with bacteria and/or increasing the targeting capacity of drugs. They may lead to an effective result with the use of less active drug substance.

Plants as antibacterial agents are used in the traditional medicine of different cultures. *O. acanthium* L. is an important plant widely used for its bactericidal properties. Although there are several studies in the literature proving the antibacterial properties of *O. acanthium* L., there is no study in which *O. acanthium* L. was encapsulated for oral use.

In this study, OAE–PLGA NPs were developed for use as a controlled drug system for oral administration against *S. aureus*. The antibacterial activity of OAE encapsulated with PLGA polymer was low compared to that of free OAE. However, the Ames and MTT tests revealed that free OAE was toxic. The mutagenicity and cytotoxicity of free OAE were eliminated after coating with PLGA. The results indicate that the PLGA NPs system improves the biocompatibility of free OAE and could be a useful approach for oral delivery against *S. aureus*. Our docking analysis results show that Quercetin in the OAE extract performed strong hydrogen bonding interactions with ASN151 and ARG187 residues in the catalytic pocket of *S. aureus MurE*, and these interactions are important interactions for enzyme inhibition. The MD simulation analysis also provides a study of the binding mode of the quercetin-based inhibitor against the *S. aureus MurE* enzyme. Quercetin formed hydrogen bond interactions with catalytic pocket binding residues LYS114, ASN151, THR152 and LYS219, which are crucial for the inhibition mechanism of *S. aureus MurE*.

## Supplemental Information

10.7717/peerj.15523/supp-1 Supplemental Information 1 Raw data.

10.7717/peerj.15523/supp-2 Supplemental Information 2 Supplemental Figure and Tables.

## Additional Information and Declarations

### Data Availability

The following information was supplied regarding data availability: The raw data is available in the Supplemental Files.
