# Supplementary material for: In vitro and in silico evaluation of the design of nano-phyto-drug candidate for oral use against Staphylococcus aureus
Source: PeerJ. 2023 Jun 8;11:e15523. doi: 10.7717/peerj.15523 (PMC10257901; doi:10.7717/peerj.15523)
Supplement: Supplemental Information 2 [file peerj-11-15523-s002.docx]

**SUPPLEMENTARY**

**Figure**


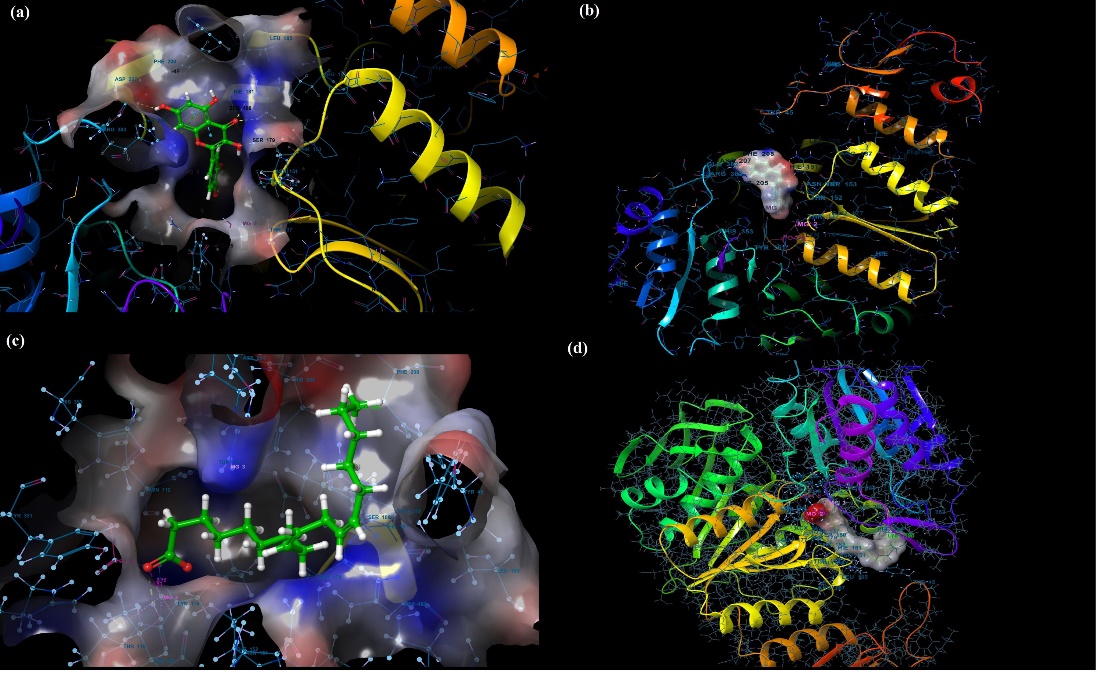


**Figure S1. (a)** and **(b)** show the molecular electrostatic potential surface of the binding pocket of the *S. aureus* MurE receptor and ligands for quercetin **(c)** and linoleic acid **(d)**

**Tables**

**Table S1.** The docking score energies and probable interaction of major compounds in *O. acanthium* against *S. aureus*

| ***Receptor***  ***PdbID: 4C13*** | ***Ligand-1***  ***Quercetin*** | ***Ligand-2***  ***Linoleic*** |
| --- | --- | --- |
| ***Docking Score(Kcal/mol)*** | -6.770 | -2.734 |
| ***H.Bond (Angstrom)*** | ASP207…….....OH(2.03)  ARG187………O(2.14,2.20)  ASN151………OH(2.71)  LYS114………OH(2.12) | THR137……..O(1.83) |
| ***Pi-Pi stacking*** | HIE181 | - |
| ***Pi-cation*** | ARG383 | - |
| ***Salt Bridge*** | - | MG……..O(2.16) |
| ***Hydrophobic Residues*** | TYR45  PHE208  TYR351 | TYR45  PHE208  TYR351  LEU185 |
| ***Polar Residues*** | THR137  THR153  THR152  ASN151  SER179  SER180  HIE181  HIS205 | THR137  THR153  THR152  ASN151  SER179  HIE181  THR115  THR111  HIS353  HIS205 |
| ***Charged (positive) Residues*** | ARG187  ARG383 | ARG187  ARG383  ARG335 |
| ***Charged (negative) Residues*** | ASP207 | ASP207  GLU177 |

**Table S2**. The calculated ADME profiles of major compounds in *O. acanthium*

| **Principal Descriptors** | ***Ligand-1***  ***Quercetin*** | ***Ligand-2***  ***Linoleic*** | **(Range 95% of Drugs)** |
| --- | --- | --- | --- |
| **Solute Molecular Weight** | 302.240 | 280.450 | (130.0 /725.0) |
| **Solute Total SASA** | 515.586 | 700.154 | (300.0 /1000.0) |
| **Solute Hydrophobic SASA** | 0.000 | 561.980 | (0.0 /750.0) |
| **Solute Hydrophilic SASA** | 287.589 | 98.729 | (7.0 /330.0) |
| **Solute Carbon Pi SASA** | 227.997 | 39.445 | (0.0 /450.0) |
| **Solute Weakly Polar SASA** | 0.000 | 0.000 | (0.0 /175.0) |
| **Solute Molecular Volume (A^3)** | 863.099 | 1189.403 | (500.0 /2000.0) |
| **Solute vdW Polar SA (PSA)** | 143.252 | 48.948 | (7.0 /200.0) |
| **Solute No, of Rotatable Bonds** | 5.000 | 14.000 | (0.0 /15.0) |
| **Solute as Donor -Hydrogen Bonds** | 4.000 | 1.000 | (0.0 /6.0) |
| **Solute as Acceptor-Hydrogen Bonds** | 5.250 | 2.000 | (2.0 /20.0) |
| **Solute Globularity (Sphere = 1)** | 0.850 | 0.775 | (0.75/0.95) |
| **Predictions for Properties:** | | | |
| **QP Polarizability (Angstroms^3)** | 27.354M | 32.833M | (13.0 /70.0) |
| **QP log P for hexadecane/gas** | 10.698M | 10.541M | (4.0 /18.0) |
| **QP log P for octanol/gas** | 19.589M | 12.348M | (8.0 /35.0) |
| **QP log P for water/gas** | 14.394M | 2.690M | (4.0 /45.0) |
| **QP log P for octanol/water** | 0.350 | 5.786 | (-2.0 /6.5) |
| **QP log S for aqueous solubility** | 2.854 | -6.043 | (-6.5 /0.5) |
| **P log S-conformation independent** | -4.043 | -4.070 | (-6.5 /0.5) |
| **QP log K hsa Serum Protein Binding** | -0.349 | 0.719 | (-1.5 /1.5) |
| **QP log BB for brain/blood** | -2.390 | -1.375 | (-3.0 /1.2) |
| **No, of Primary Metabolites** | 5 | 4 | (1.0 /8.0) |
| **Predicted CNS Activity (-- to ++)** | -- | -- |  |
| **HERG K+ Channel Blockage: log IC50** | -5.042 | -3.547 | (concern below -5) |
| **Apparent Caco-2 Permeability (nm/sec)** | 18 | 290 | (<25 poor, >500 great) |
| **Apparent MDCK Permeability (nm/sec)** | 6 | 165 | (<25 poor, >500 great) |
| **QP log Kp for skin permeability** | -5.537 | -1.856 | (Kp in cm/hr) |
| **Jm, max transdermal transport rate** | 0.001 | 0.004 | (micrograms/cm^2-hr) |
| **Lipinski Rule of 5 Violations** | 0 | 1 | (maximum is 4) |
| **Jorgensen Rule of 3 Violations** | 1 | 1 | (maximum is 3) |
| **% Human Oral Absorption in GI (+-20%)** | 52 | 92 | (<25% is poor) |
| **Qual, Model for Human Oral Absorption** | Medium | HIGH | (>80% is high) |
